# Prophylactic Moxibustion in Preventing Postoperative Urinary Retention of Hemorrhoidectomy: A Study Protocol for a Randomized Controlled Trial

**DOI:** 10.3389/fsurg.2022.898097

**Published:** 2022-07-05

**Authors:** Lijiang Ji, Aihua Wang, Qian Fan, Naijin Zhang, Liping Weng, Jing Gu

**Affiliations:** ^1^ Department of Anorectal Surgery, Changshu Hospital Affiliated to Nanjing University of Chinese Medicine, Changshu, China; ^2^ Rheumatology and Immunology Department, First Teaching Hospital of Tianjin University of Traditional Chinese Medicine, Tianjin, China; ^3^ Graduate School, Tianjin University of Traditional Chinese Medicine, Tianjin, China

**Keywords:** hemorrhoidectomy, postoperative urinary retention, moxibustion, prevention, protocol

## Abstract

**Backgrounds:**

Postoperative urinary retention (POUR) is one of the most common complications after hemorrhoidectomy. The best treatment for POUR is prevention and should be involved in the whole perioperative period. Moxibustion has been used to treat urinary retention for thousands of years, and clinical studies have also proved its effects. We try to carry out a randomized, controlled, prospective study to observe whether prophylactic moxibustion could effectively reduce the incidence of POUR of hemorrhoidectomy in 24 h.

**Methods:**

This study is a single-center, evaluator-blinded, randomized, and controlled trial. Participants who meet the inclusion and exclusion criteria in this RCT will be randomly assigned to either the treatment group (moxibustion) or the control group (tamsulosin hydrochloride) in a 1:1 ratio according to a computer-generated randomization list. Both moxibustion and tamsulosin will be used 1, 10, and 24 h after operation, respectively. The outcomes of occurrence of POUR, time to first urination, catheterization rate, urinary tract infection, length of hospitalization, and adverse effects will be recorded.

**Discussion:**

The findings of the study will help to explore the preventive efficacy of prophylactic moxibustion against POUR of hemorrhoidectomy in 24 h.

**Trial Registration:**

CHiCTR, CHiCTR2000039350, registered 24 October, 2020, http://www.chictr.org.cn/showproj.aspx?proj = 63204.

## Background

The optimal treatment for circumferential mixed hemorrhoids is surgery (1, 2), and urinary retention is one of the most common complications after hemorrhoidectomy, with an incidence of 32.8% (3) or even higher. Mostly, postoperative urinary retention (POUR) is caused by anesthesia and analgesia, intravenous fluids, mental tension, and duration of surgery. POUR can easily cause urinary tract infections and secondary reflux nephropathy, which increases the suffering of patients and prolongs their recovery (4).

Catheterization is the main effective method for resolving POUR, but it may lead to urethral injury and stricture to increase the predisposition for urinary tract infections (UTIs). After the withdrawal of the catheter, the patients may also suffer from POUR, which could increase the cost of the hospitalization period. Therefore, the best treatment for POUR is prevention and should be involved in the whole perioperative period (5–7). The methods of controlling intraoperative intravenous fluid volume, taking a slow time to ambulate, and systemic opioids after the surgery are commonly used.

Several studies have demonstrated the benefit of prescribing alpha-blockers (prazosin (8), tamsulosin (9)) in preventing POUR (10), but these may cause many side effects such as headache and positional hypotension. In China, moxibustion is a commonly used method for urinary retention clinically. It is a traditional Chinese medicine therapy, and it plays an important role in the traditional medical systems of China against urinary incontinence, dysmenorrhea, knee osteoarthritis, soft tissue injury, heel pain, asthma, and urinary retention (11). Until now, there have been many studies on the effects of moxibustion on the human body (12–14), and clinical studies also proved the curative effect of moxibustion against POUR (15, 16). Mechanism studies have reported its thermal effects, radiation effects, and pharmacological actions to function as an analgesic, alleviating inflammation and enhancing immunity.

In our group, we tried to preliminarily perform prophylactic moxibustion on patients with prolapsed hemorrhoids right after surgery and found that it might be an effective way to reduce the incidence of POUR in 24 h. Therefore, we try to carry out a randomized controlled study with the following objectives: (1) to observe whether prophylactic moxibustion could effectively reduce the occurrence of POUR of severe prolapsed hemorrhoids in 24 h and (2) to provide evidence for promoting the application of moxibustion to prevent POUR. Tamsulosin will be used as an intervention in the control group because of its relatively more amenable side effects and greater patient satisfaction, as reported (17, 18).

## Methods

### Study Design

The study is a single-center, evaluator-blinded, randomized controlled trial (RCT). The protocol has been approved by the Ethics Committee of Changshu Hospital Affiliated to Nanjing University of Chinese Medicine, and the trial will be carried out in accordance with the Declaration of Helsinki. The protocol conforms to the Standard Protocol Recommendations for Interventional Trials (SPIRIT) 2013 Statement (19), and the results will be reported according to the CONSORT Statement extension for trials (20), the STandards for Reporting Interventions in Clinical Trials of Acupuncture (STRICTA) (28), and the STandards for Reporting Interventions in Clinical Trials of Moxibustion (STRICTOM). The clinical trial has been registered in CHiCTR (No.CHiCTR2000039350). The schedule of enrollment, interventions, and assessments is given in Table 1.

**Table 1:** Schedule of enrollment, intervention, and assessments.

Time point	Enrollment	Treatment phase		
		1 h	10 h	24 h
**Enrollment**				
Eligibility screen	√			
Informed consent	√			
demographics	√			
History of disease	√			
Allocation	√			
Intervention				
Moxibustion		√	√	√
Tamsulosin		√	√	√
Assessments				
POUR incidence				√
Time to first urination				√
Catheterization rate				√
Incidence of UTI				√
Length of hospitalization				√
Adverse effects				√

### Participants

**Figure 1:**
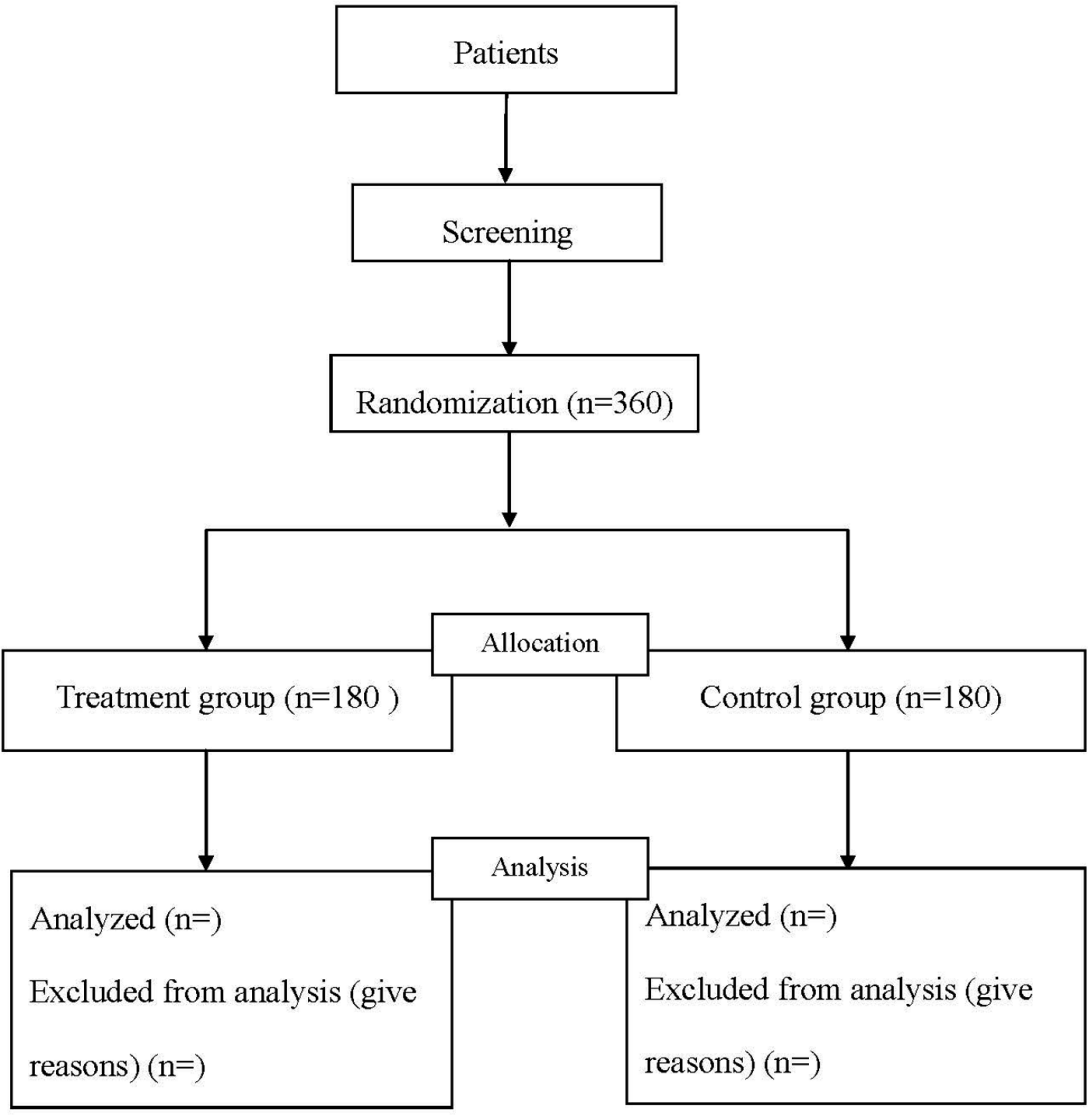
Flow diagram of the study.

Participants will be recruited from inpatients of the Proctology Department of Changshu Hospital Affiliated to Nanjing University of Chinese Medicine, and they will be randomly assigned to either the treatment group or control group in a 1:1 ratio (Figure 1). The patients will be recruited from inpatients via posters in the department from March 01, 2021.

### Inclusion Criteria

Patients will be included if they meet the following inclusion criteria:

(1) meet the diagnostic criteria for circumferential mixed hemorrhoids, with internal hemorrhoids grade III–IV;
(2) aged from 18 to 70 years;
(3) without obvious deformity of the anus before the operation; and
(4) with written informed consent.

### Exclusion Criteria

Patients will be excluded if they present the following exclusion criteria:

(1) have undergone mixed hemorrhoids surgery or combined with other operations;
(2) women in pregnancy, lactation, and menstruation;
(3) with rectal cancer, rectal polyps, tuberculosis, Crohn's disease, and other anorectal diseases;
(4) with glucose-6-phosphate dehydrogenase (G6PD) deficiency, inadequate conditions of butyrylcholinesterase enzyme, methemoglobinemia, and myasthenia gravis;
(5) with severe respiratory, digestive, cerebrovascular disease, and obvious abnormal liver and kidney functions;
(6) with urinary system diseases such as prostate hyperplasia, neurogenic bladder, urethral injury, urethral stricture; and
(7) with ulcers, injuries, infections, scars, and allergies in the abdomen.

### Sample Size

The sample size estimate is based on a two-arm normal design. The ratio of the two groups is set as 1:1, and the significance level of the test is targeted at 0.05, with the power achieved to detect a difference set as 0.8. According to a previous literature report, the incidence of POUR after taking tamsulosin hydrochloride was 28.1% (21). In our preliminary experiment, the incidence of POUR after moxibustion therapy is 15%. Considering the dropout rate of no more than 20%, 180 cases will be required for each group.

### Randomization

Patients will be randomly divided into a treatment group (moxibustion) and a control group (tamsulosin hydrochloride) in a 1:1 ratio. The randomization sequence will be generated by an independent statistician not involved in the statistical analysis with SPSS 22.0 software. The random numbers will be placed in opaque, sealed envelopes and kept in a safe place until the study is complete.

### Blinding

All patients will be informed to be equally allocated to the treatment group and control group before enrollment. Blinding of moxibustion is quite difficult to achieve. The treatment will be performed by trained and experienced moxibustion members. Surgeons, moxibustion practitioners, and patients will not be blinded due to the limitation of treatment, while outcome assessors and statisticians will be blinded to treatment allocation.

### Interventions

The same operation (tissue selecting technique with a mega-window stapler combined with anal canal epithelial preservation operation), anesthesia, and intraoperative and postoperative management will be performed for patients in both groups. Subarachnoid anesthesia will be applied with 10 mg of Naropin at L3–4. The intraoperative fluid is 500 ml, and the postoperative fluid is 500 ml for anti-infection and hemostasis.

### Treatment Group

Moxibustion therapy will be used 1, 10, and 24 h after operation. Moxibustion members who have more than 1-year experience in mild moxibustion will be retrained for standard management of the study. The moxa rolls for moxibustion are produced by Nanyang Chinese Medicine Airong Co. Ltd., in 18 × 200 mm. Points of Qihai (CV 6), Zhongji (CV 5), Guanyuan (CV 4), and bilateral Sanyinjiao (SP 6) will be selected for moxibustion.

In the procedure of moxibustion, the patient takes the supine position with the abdominal skin exposed; the moxibustion members lit the moxa rolls and put them 2–3 cm above the five points for 20 min. When the patients cannot urinate with urgent micturition desire within 24 h after the operation, catheterization will be performed.

### Control Group

Tamsulosin hydrochloride [0.2 mg, Astellas Pharmaceutical Co., Ltd., China] will be given once to patients 1, 10, and 24 h after operation. When the patients could not urinate with urgent micturition desire within 24 h after the operation, catheterization will be performed (Table 1).

### Outcome Variables

#### Primary Outcomes

*POUR incidence*: POUR is deﬁned as any of the following (21, 22): (1) patient discomfort, a sensation of a full bladder, palpable, distended bladder, with a volume of urine 400 ml; (2) any estimated postvoid residual (PVR) volume of urine greater than or equal to 200 ml per bladder scan (21); and (3) need for catheterization in 24 h.

Patients are requested to freely report the sensation of a full bladder, palpable, distended bladder at any time after the operation. The occurrence of POUR in 24 h after the operation is recorded, and POUR incidence = the number of patients who meet the diagnosis of POUR/total number of included cases in each group.

#### Secondary Outcomes

(1) Time to first urination: the time from the end of the operation to the first urination.
(2) Catheterization rate: the number of people who need catheterization in 24 h after operation in each group, catheterization rate = number of catheterization people/number of people included in each group.
(3) Incidence of urinary tract infection (UTI): the occurrence of urinary tract infection during hospitalization, rate = number of infections/number of people included in each group.
(4) Length of hospitalization: The average length of hospitalization of patients.
(5) Adverse effects: Potential AEs may occur during the study. Patients may suffer from empyrosis, itching, allergy, dizziness with moxibustion and orthostatic hypotension, headache, dizziness, and vomiting with tamsulosin. The supervisor will manage the intervention, and any suspected AEs will be discussed with the principal investigator of the project team. If the patient shows any discomfort or changes in their condition, they are instructed to inform the investigators. The physician will provide the necessary treatment to alleviate any AEs.

### Discontinue and Data Monitoring

Patients will be dropped if they are with the following conditions: (1) With severe adverse events (AEs); (2) Unwilling to continue the study protocol. The supervisor will make a decision to discontinue if more than 25% of the patients discontinue intervention due to AEs. Case report forms (CRFs) will be used in data collection to record demographics, assessments, and reasons for patient dropout. All CRFs will be stored in a locked cabinet. At the end of the study, the investigator will submit the CRFs to the data management committee, and the investigators cannot modify the data. The data monitoring committee is independently chaired by the Statistics Teaching and Research Office of Changshu Hospital Affiliated to Nanjing University of Chinese Medicine and claims no conflict of interest. All CRFs will be preserved for at least 5 years after publication, and access to the original data of readers and reviewers is available from the corresponding author on reasonable requests.

### Statistical Methods

Full analysis set (FAS) and per-protocol set (PPS) will be both used for statistical analysis in this study. In FAS, for missing outcome data, multiple imputations will be used o. For continuous data, they will be represented as mean ± standard deviation and frequency or percentage for categorical data. Statistics will be analyzed with SPSS 22.0 (SPSS Inc., Chicago, IL). For primary outcomes, the chi-square test will be applied to analyze the POUR incidence. For secondary outcomes, the comparison of UTI incidence, AEs, and catheterization rate will be analyzed by the chi-square test; the independent-sample *t*-test or Mann–Whitney *U* test for intergroup comparison will be used for the time to first urination and the length of hospitalization after considering the normality and homogeneity. The dropout rates will be analyzed by the chi-square test, and the impact of the dropout rate will be further analyzed by comparing dropout reasons, characteristics of dropout cases between groups, and the results using intention-to-treat and per protocol. Statistical testing is two-sided, and *p* < 0.05 is considered statistically significant.

### Oversight and Monitoring

The Office of Academic Research in our hospital will supervise and monitor the trial independently and audit the trial twice a year. If any changes are needed to the protocol, we will first notify the sponsor. After making the revised protocol, we will update it on the registry site.

## Discussion

Many risk factors in anorectal surgery, such as anesthesia, surgical stimulation, or pain, can easily cause spasms of the bladder neck and urethral sphincter or weak contraction of the bladder detrusor, inducing reflex dysuria and a high incidence of POUR. POUR is a common urological emergency, which occurs 6–8 h after hemorrhoidectomy, manifested with urgency, lower abdominal pain, distress, refusal to press, and bladder enlargement. Therefore, how to effectively prevent POUR after hemorrhoidectomy is important.

Tamsulosin hydrochloride is a superselective *α*-1a receptor blocker, and it could block *α*1a adrenergic receptors in the prostate and relax the smooth muscle of the prostate, thus improving dysuria and other symptoms. The incidence of postural hypotension is relatively low due to the high selectivity of this drug. It can not only effectively treat urinary retention, and could also reduce the incidence of POUR (23, 24), yet with certain side effects, including dizziness, headache, syncope, and retrograde ejaculation (25).

In China, moxibustion is a commonly used therapy for urinary retention. Moxibustion is an external treatment based on TCM theory. By baking acupoints with burning moxa wool, it is characterized by simple operation, painless, fewer side effects, and stable efficacy. It has been used for more than 2,500 years, and it could dredge meridians and regulate qi-blood to prevent and cure diseases according to TCM theory. By burning moxa without flame over the acupoints, patients will get a warm feeling when close to the body. Its thermal effects, radiation effects, and pharmacological actions (26) could function as an analgesic, alleviating inflammation and enhancing immunity.

Clinical studies have proved that moxibustion could effectively improve urination disorder (27–29), yet there are few highly designed clinical trials to prove its effects in preventing POUR in hemorrhoidectomy. Therefore, we designed a randomized controlled clinical study to compare the difference of prophylactic moxibustion with tamsulosin in reducing the incidence of POUR against patients of hemorrhoidectomy in 24 h and to clarify the efficacy of moxibustion to prevent POUR.

A double-blinded, placebo-controlled trial is supposed to be more applicable to assess the therapeutic effect of prophylactic moxibustion in reducing POUR, but it is difficult to carry out sham moxibustion because of the nature of the moxibustion procedure. Heat is supposed to be the most important factor of moxibustion, and the warm stimulation of moxibustion could be easily distinguished from sham moxibusiton. Although there are several reports about sham moxibustion devices used in clinical trials (30–32), it cannot fully be achieved. Thus, double-blinding and placebo control will not be involved in this study. However, many Chinese patients are awaiting to have a positive effect on moxibustion, despite the magnitude of the effect. It made the placebo effect an inseparable part of moxibustion efficacy. It has been previously discussed that there might be placebo effects involved in intervention with moxibusion (30, 31). When analyzing the results, the placebo effect should be taken into consideration in our study.

In addition, there are still several other limitations in this study. First, our study is single-centered, which may affect the conclusions. Second, many studies reported that the dosage of tamsulosin is higher with 0.4 mg (33, 34) or 0.8 mg (35). We used 0.2 mg tamsulosin according to the instruction in this study, and it might influence the effect.

## Trial Status

This protocol is version 3.0. 2021-9-28, and the study is still ongoing.

## Ethics Statement

The studies involving human participants were reviewed and approved by Ethics Committee of Changshu Hospital Affiliated to Nanjing University of Chinese Medicine. The patients/participants provided their written informed consent to participate in this study.
